# Elevated Admission Base Deficit Is Associated with a Complex Dynamic Network of Systemic Inflammation Which Drives Clinical Trajectories in Blunt Trauma Patients

**DOI:** 10.1155/2016/7950374

**Published:** 2016-11-15

**Authors:** Othman Abdul-Malak, Yoram Vodovotz, Akram Zaaqoq, Jesse Guardado, Khalid Almahmoud, Jinling Yin, Brian Zuckerbraun, Andrew B. Peitzman, Jason Sperry, Timothy R. Billiar, Rami A. Namas

**Affiliations:** ^1^Department of Surgery, University of Pittsburgh, Pittsburgh, PA 15213, USA; ^2^Center for Inflammation and Regenerative Modeling, McGowan Institute for Regenerative Medicine, University of Pittsburgh, Pittsburgh, PA 15219, USA; ^3^Department of Critical Care Medicine, University of Pittsburgh, PA 15213, USA

## Abstract

We hypothesized that elevated base deficit (BD) ≥ 4 mEq/L upon admission could be associated with an altered inflammatory response, which in turn may impact differential clinical trajectories. Using clinical and biobank data from 472 blunt trauma survivors, 154 patients were identified after excluding patients who received prehospital IV fluids or had alcohol intoxication. From this subcohort, 84 patients had a BD ≥ 4 mEq/L and 70 patients with BD < 4 mEq/L. Three samples within the first 24 h were obtained from all patients and then daily up to day 7 after injury. Twenty-two cytokines and chemokines were assayed using Luminex™ and were analyzed using two-way ANOVA and dynamic network analysis (DyNA). Multiple mediators of the innate and lymphoid immune responses in the BD ≥ 4 group were elevated differentially upon admission and up to 16 h after injury. DyNA revealed a higher, sustained degree of interconnectivity of the inflammatory response in the BD ≥ 4 patients during the initial 16 h after injury. These results suggest that elevated admission BD is associated with differential immune/inflammatory pathways, which subsequently could predispose patients to follow a complicated clinical course.

## 1. Introduction

Traumatic injury can result in sudden metabolic and physiological alterations that are frequently proportionate to the degree and magnitude of the injury sustained [1–3]. Consequently, the host's homeostatic mechanisms—often driven or accompanied by a broad systemic inflammatory response—attempt to correct for these metabolic perturbations and restore normal body functions [4, 5]. However, with significant disruption of the homeostatic mechanism and inability to compensate for these metabolic changes, cellular hypoperfusion and metabolic acidosis ensue [6, 7]. Further complicating the clinical scenario is the extent of the perpetuated metabolic derangement that could propagate the trauma-induced inflammatory response [4, 8–10]. This results in a positive self-feedback loop of inflammation driving damage or dysfunction, which in turn stimulates further inflammation [11–13]. We and others have hypothesized that the outcome of this vicious cycle eventually leads to immune dysregulation, which predispose trauma patients to develop complications such as multiple organ failure and nosocomial infections [14–17].

The early identification and correction of significant metabolic disturbances are one of the key components in managing critically ill patients [6, 18–20]. Blood lactate and arterial base deficit (BD) are the most commonly used circulating markers of systemic metabolic acidosis [2, 21–24]. Multiple studies have validated the usefulness of blood lactate and BD in trauma patients as markers of injury severity [25–28], as endpoints of resuscitation [20, 29], as a means to monitor treatment efficacy [30–32], and as predictors of outcome [33–36]. Despite indicating the severity of hypoperfusion [37, 38], measurement of lactate alone may not fully encompass the extent of the underlying metabolic acidosis [39]. On the contrary, BD can quantify the extent of both anaerobic and aerobic acidosis and may be a better clinical indicator for the global assessment of the metabolic acid-base status [32, 39–41]. In addition, prior studies have suggested that elevated arterial BD (≥4 mEq/L) upon admission after injury can be a reliable predictor of multiple organ dysfunction, morbidity, and mortality in moderately/severely injured patients [21, 34, 36, 42–44].

Despite the large volume of published data studying the utility of arterial BD among trauma patients, no study to date has examined the temporal effect of metabolic derangement, suggested by elevated BD, on the dynamics of the trauma-induced inflammation. In addition, it is not clear which immune phenotypes are associated with the ensuing metabolic disturbance in trauma patients who follow a complicated clinical course. To address this complexity, we analyzed clinical and inflammatory biomarker data from a cohort of 472 blunt trauma survivors. To reduce the possible impact of confounding variables, we excluded patients who received large volumes (>2 liters) of intravenous fluids or blood components* en route*, as well as patients with alcohol intoxication upon admission. After applying these exclusion criteria, we sought to derive two subgroups based on a BD cutoff of below and above 4 mEq/L.

These selection criteria yielded two subgroups of patients with admission BD < 4 versus BD ≥ 4. Plasma samples from these two groups were analyzed subsequently for dynamic changes in inflammatory mediators. Our results revealed that patients in the BD ≥ 4 group exhibited an early, differential immune profile upon admission independent of age, gender, and injury severity. In parallel to these results, data-driven modeling suggested a higher, sustained degree of activation of both innate and lymphoid pathways in the BD ≥ 4 group when compared to BD < 4 group. Collectively, these analyses suggest that BD ≥ 4 mEq/L after injury could drive differential immune pathways, which in turn may predispose trauma patients to develop adverse in-hospital outcomes.

## 2. Methods

### 2.1. Patient Enrollment and Sampling

All human sampling was carried out following approval by the University of Pittsburgh Institutional Review Board (IRB), and informed consent was obtained either from the patient or from the next of kin, as per IRB regulations. Patients who deemed eligible for enrollment were at least 18 years of age at time of the trauma, admitted to the Intensive Care Unit (ICU) as part of the posttrauma management, and were expected to survive beyond the initial 24 h after injury as per the on-call trauma surgeon. Reasons for ineligibility were isolated head injury or brain death criteria, pregnancy, and penetrating trauma. Three plasma samples, starting with the initial blood draw upon arrival, were assayed within the first 24 h following injury and then from days 1 to 7 after injury. The blood samples were centrifuged, and plasma aliquots were stored in cryopreservation tubes at −80°C for subsequent analysis of inflammatory mediators.

### 2.2. Data Collection

Demographic and clinical data including ICU length of stay (LOS), hospital LOS, days on mechanical ventilation, arterial BD, lactic acid, heart rate, systolic blood pressure, injury severity score (ISS) [45], the abbreviated injury scale (AIS) [46], Glasgow coma scale (GCS), the Marshall Multiple Organ Dysfunction Score (MODScore) [47] (which was calculated daily as described previously [17]), and the Shock Index [48] (which identifies the degree of shock in trauma patients, calculated based upon the ratio of heart rate to the systolic blood pressure, where an index > 1 signifies hypovolemic shock) were collected from the inpatient electronic medical record and the trauma registry database.

### 2.3. Study Design and Selection of Subgroups

Using a prospectively maintained clinical database and biobank of 472 blunt trauma survivors (after excluding 21 nonsurvivors) admitted or transferred to Emergency Department (ED) of the Presbyterian University Hospital (Level 1 trauma center), the salient characteristics of which were described recently [17]. Initially, we sought to reduce possible confounding factors that might affect admission BD. Prior studies have suggested that increased serum ethanol levels could alter BD values independent of other factors such as initial blood pressure, injury severity, and the amount of blood loss [49–51]. Accordingly, patients with documented alcohol intoxication (defined as serum ethanol level > 10 ng/dL) upon admission to the ED were excluded from the analysis. Another possible confounder for which we sought to control was the administration of crystalloids and blood products prior to ED admission. While crystalloids and blood products are an integral component for the management of patients in shock, administration of large volumes of crystalloids and blood components can significantly alter BD values [52–55]. Accordingly, patients who received large volumes of fluids or blood products (>2 liters)* en route* were further excluded from the analysis.

After applying these aforementioned inclusion and exclusion criteria (*see above*), we next sought to derive two subgroups of trauma patients based on admission BD values. Prior studies have suggested that an initial arterial BD value of >4 mEq/L was associated with increased patient mortality and development of organ failure [21, 43, 44]. Thus, we applied a BD cutoff value of 4 mEq/L, which segregated trauma patients into two distinct groups: BD < 4 (*n* = 70) and BD ≥ 4 (*n* = 84). The subgroups were then subsequently analyzed for differences in clinical outcome, development of organ dysfunction, disposition, inflammatory biomarker trajectories, and dynamic inflammatory network connectivity.

### 2.4. Inflammation Biomarker Analysis

Blood samples were collected into citrated tubes via peripheral venous, central venous, or arterial catheterization within 24 h of admission and daily up to 7 days after injury. The blood samples were centrifuged, and plasma was stored in multiple aliquots at −80°C for subsequent analysis of inflammatory mediators. The human inflammatory MILLIPLEX™ MAP Human Cytokine/Chemokine Panel-Premixed 24 Plex, MILLIPLEX MAP Human Th17 Panel (Millipore Corporation, Billerica, MA), and Luminex 100 IS analyzer (Luminex, Austin, TX) were used to measure plasma levels of interleukin 1*β* (IL-1*β*), IL-1 receptor antagonist (IL-1RA), IL-2, soluble IL-2 receptor-*α* (sIL-2R*α*), IL-4, IL-5, IL-6, IL-7, IL-8 (CCL8), IL-10, IL-13, IL-15, IL-17A, interferon *γ* (IFN-*γ*), IFN-*γ* inducible protein 10 (IP-10) (CXCL10), monokine induced by gamma interferon (MIG; CXCL9), macrophage inflammatory protein 1*α* (MIP-1*α*) (CCL3), MIP-1*β* (CCL4), monocyte chemotactic protein 1 (MCP-1) (CCL2), granulocyte-macrophage colony stimulating factor (GM-CSF), Eotaxin (CCL11), and tumor necrosis factor alpha (TNF-*α*). The Luminex system was used in accordance to the manufacturer's instructions.

### 2.5. Statistical Analysis

All data were analyzed using SigmaPlot™ 11 software (Systat Software, Inc., San Jose, CA) and GraphPad PRISM (GraphPad Software, Inc., La Jolla, CA). Statistical difference between subgroups was determined by either Mann–Whitney* U* test, Chi-square, or Hazard Ratio as appropriate. Group-time interaction of plasma inflammatory mediators' levels was determined by two-way analysis of variance (ANOVA). To quantify the overall production of the statistically significant mediators, we calculated the area under the curve (AUC) using the mean values for each time point in a given time frame and then calculated the fold change difference between the two subgroups. *P* < 0.05 was considered statistically significant for all analyses.

### 2.6. Data-Driven Modeling: Dynamic Network Analysis (DyNA)

The goal of this analysis was to gain insights into the temporal dynamic changes in network interconnectivity and complexity of the posttraumatic inflammatory response between the BD < 4 and BD ≥ 4 subgroups. The mathematical formulation of this method is to calculate the correlation among inflammatory mediators by which we can examine their dependence across time. To do so, inflammatory mediator networks were created in adjacent 8 h time periods over the first 24 h (0–8 h, 8–16 h, and 16–24 h) using MATLAB® (The MathWorks, Inc., Natick, MA) as we have done previously [17, 56, 57]. Connections in the network were created if the correlation coefficient between two nodes (inflammatory mediators) was greater or equal to a threshold of 0.7. For the network density calculation, in order to account for network sizes (number of significantly altered nodes) in the adjacent 8–h time periods detailed above, we utilized the following formula:

(1) Network  Density=Total  number  of  egdes×Number  of  total  nodesMaximum  possible  edges  among  total  nodes.

## 3. Results

### 3.1. Demographics and Clinical Outcomes of Study Subgroups

The overall study cohort consisted of 472 blunt trauma survivors who were admitted to the ICU after being resuscitated, which was described recently [17]. Of these patients and based on selection criteria (see Section 2), we identified 70 patients with an admission BD < 4 mEq/L (age: 49 ± 2; male/female [M/F]: 55/15; ISS: 21 ± 1; and GCS: 13 ± 0.5) and 84 patients with an admission BD ≥ 4 mEq/L (age: 46 ± 2; M/F: 55/29; ISS: 23 ± 1; and GCS: 13 ± 0.6). Overall, males were predominant in both BD < 4 and BD ≥ 4 groups (78.6% and 65.5% resp.; *P* = 0.07) with no statistical difference in mean age (*P* = 0.3) between the two subgroups. In addition, both BD < 4 and BD ≥ 4 groups sustained trauma in different forms, with motor vehicle crashes being the predominant mechanism of injury (67% and 65%, resp.). There was no statistical difference in the mechanism of injury (*P* = 0.9) and ISS (*P* = 0.1) between the two subgroups. Importantly, the ICU LOS (*P* = 0.005), hospital LOS (*P* < 0.001), and days on mechanical ventilation (*P* = 0.02) were all statistically significantly longer in the BD ≥ 4 group when compared to the BD < 4 group (see Table 1).

### 3.2. Biochemical and Physiological Parameters

**Figure 1:**
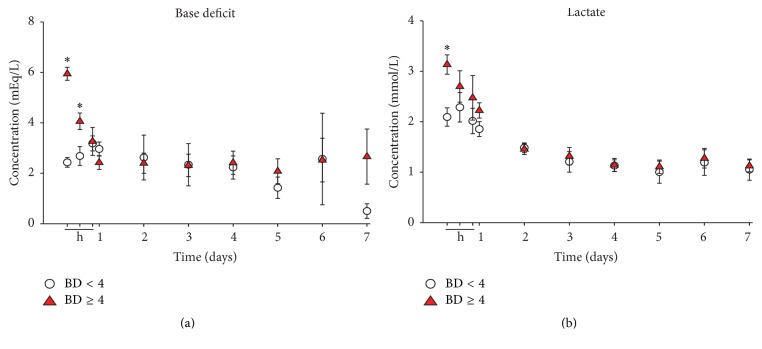
(a) Arterial base deficit (BD) from time of admission up to 7 days after injury between BD ≥ 4 and BD < 4 subgroups. The BD ≥ 4 group had statistically significantly elevated BD levels at 8 h and 16 h after injury when compared to the BD < 4 group. BD in both subgroups approach similar values at 24 h after injury. ^*∗*^ *P* < 0.05 by two-way ANOVA. (b) Plasma lactate levels from time of admission up to 7 days after injury between BD ≥ 4 and BD < 4 subgroups. The BD ≥ 4 group had statistically significantly elevated lactate levels at 8 h after injury when compared to the BD < 4 group. BD in both subgroups approaches normal values at 24 h after injury. ^*∗*^ *P* < 0.05 by two-way ANOVA.

Since our study subgroups were selected based on admission BD values, we next sought to examine time course changes in BD across multiple time points within the first 24 h and up to day 7 after injury. As expected per our study design, the admission BD was statistically significantly elevated at 8 h after injury in the BD ≥ 4 group when compared to the BD < 4 group (6.7 ± 0.4 versus 1.7 ± 0.1 resp.; *P* < 0.001; Figure 1(a)). Although these elevations persisted up to 16 h after injury, BD values in the BD ≥ 4 group declined significantly at 24 h, reaching levels similar to those of the BD < 4 group from days 1 to 7 after injury (Figure 1(a)). Given that BD is a surrogate marker of lactic acid accumulation and hence metabolic acidosis [53], we next sought to determine venous lactate levels at the corresponding time points in which BD values were assessed. This analysis revealed that the BD ≥ 4 group had statistically significantly higher lactate levels upon admission when compared to the BD < 4 group (3.4 ± 0.3 versus 2.1 ± 0.1 resp.; *P* < 0.001; Figure 1(b)). Thereafter, lactate levels declined steadily within 24 h of injury reaching normal ranges by day 2 and up to day 7 after injury (Figure 1(b)).

**Figure 2:**
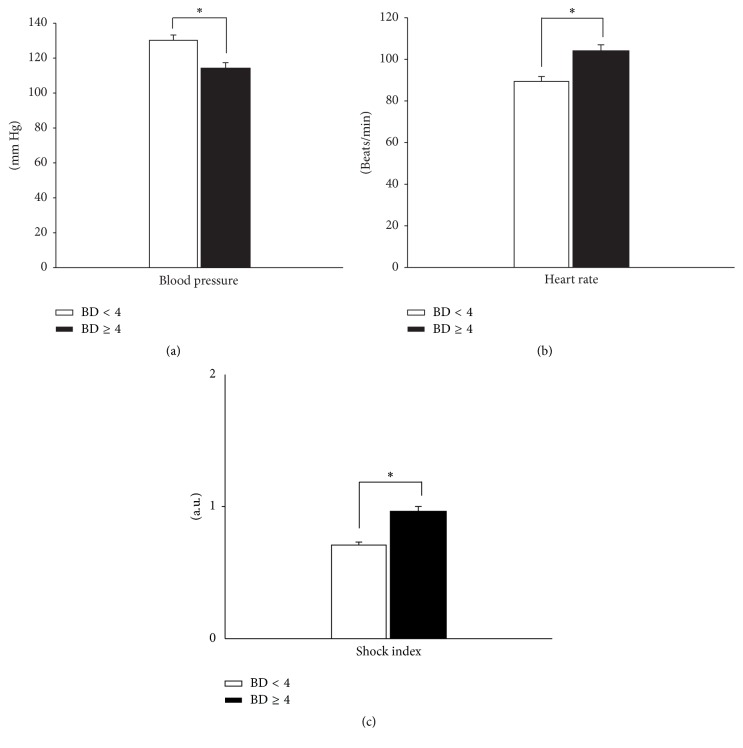
(a) Differences in (a) admission systolic blood pressure (SBP), (b) admission heart rate (HR), and (c) Shock Index between BD ≥ 4 and BD < 4 subgroups. (a) Admission SBP, (b) admission HR, and (c) Shock Index (HR/SBP) were statistically significantly different in the BD ≥ 4 group when compared to the BD < 4 group. ^*∗*^ *P* < 0.05 by Mann–Whitney* U* test.

Vital signs are an integral part of the initial cache of parameters used to assess the degree of shock. Current standards of care resuscitation guidelines following trauma or hemorrhagic shock are almost exclusively based on parameters such as the systolic blood pressure (SBP), heart rate (HR), and the mean arterial pressure (MAP) [58–60]. Analysis of these physiological parameters showed that the BD ≥ 4 group had a statistically significantly lower mean admission SBP when compared to the BD < 4 group (114 ± 3 mmHg versus 130 ± 3 mmHg resp.; *P* < 0.001; Figure 2(a)). In addition, the BD ≥ 4 group had a statistically significantly higher mean HR when compared to the BD < 4 group (104 ± 3 bpm versus 89 ± 2 bpm resp.; *P* < 0.001; Figure 2(b)). However, there was no statistically significant difference (*P* = 0.78) with regard to the proportion of patients with overt hypotension (defined as MAP < 60 mmHg) upon admission in the BD ≥ 4 group (5/84 [6%]) when compared to the BD < 4 group (2/70 [3%]).

Next, we sought to evaluate the degree of shock between the two subgroups by calculating the Shock Index (SI; see Section 2). The BD ≥ 4 group had statistically significantly higher degree of shock when compared to BD < 4 group (SI: 0.96 ± 0.03 versus 0.7 ± 0.02, resp.; *P* < 0.001; Figure 2(c)). We suggest that these physiological differences between the two subgroups could reflect the hypoperfusion status occurring at the tissue level in otherwise apparently stable trauma patients.

### 3.3. Body Region Involvement and Development of Multiple Organ Dysfunction

**Figure 3:**
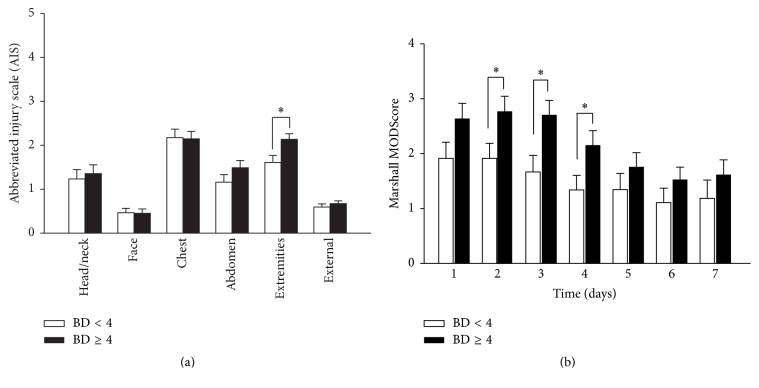
(a) Abbreviated injury scale (AIS) analysis of injury patterns in BD ≥ 4 and BD < 4 subgroups. The BD ≥ 4 group exhibited greater extremity injury when compared to the BD < 4 group. ^*∗*^ *P* < 0.05 by Mann–Whitney* U* test. (b) Multiple Organ Dysfunction Score (MODScore) in BD ≥ 4 and BD < 4 subgroups from days 1 through 7 after injury. The BD ≥ 4 group had a statistically significantly higher degree of organ dysfunction from days 2 to 4 after injury compared to the BD < 4 group. ^*∗*^ *P* < 0.05 by two-way ANOVA.

Although the ISS was not statistically significantly different between the two subgroups, an analysis of the AIS revealed a statistically significantly (*P* < 0.05) higher degree of extremity injury in the BD ≥ 4 group when compared to BD < 4 group (Figure 3(a)). In addition, the BD < 4 and BD ≥ 4 subgroups differed in their degree of multiple organ dysfunction (MOD), as indicated by the Marshall MODScore [47], which was calculated at each relevant time point in which inflammation biomarkers were assessed. This analysis suggested that the BD ≥ 4 group had a persistently elevated mean MODScore from day 1 through day 7 after injury, being statistically significantly higher (*P* < 0.001) at day 2 through day 4 when compared to BD < 4 group (Figure 3(b)).

### 3.4. Inpatient Management and Hospital Discharge

Blood and blood components are integral part of management in trauma patients who present with clinically observable hypotension (SBP < 90 mmHg) or hypovolemic shock refractory to crystalloid resuscitation. Analysis of blood transfusion requirement revealed that 43/84 patients (51%) of the BD ≥ 4 group required blood transfusion in the first 24 h after injury as compared to 22/70 patients (31%) in the BD < 4 group (Table 1). This analysis suggested that hypotensive patients with admission BD ≥ 4 mEq/L had a 1.7-fold risk for requiring blood transfusion (*P* = 0.012) within the initial 24 h after injury.

**Figure 4:**
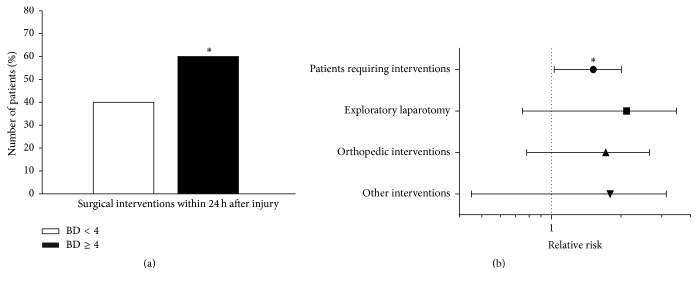
(a) Percentage of patients requiring urgent surgical interventions upon discharge from the trauma bay. 61% of patients in the BD ≥ 4 group required immediate surgical interventions compared to 41% in the BD < 4 group. ^*∗*^ *P* < 0.05 by Chi-square. (b) Relative risk of requiring operative management in the first 24 h after trauma. Having an admission BD ≥ 4 is associated with a 1.5-fold risk of requiring surgical interventions in the first 24 h after injury. ^*∗*^RR = 1.5; 95% CI [1.056–2.033]; *P* < 0.05.

The decision to perform urgent surgical interventions after trauma is based on a combination of clinical parameters and diagnostic tests that are performed in the ED/trauma bay [60, 61]. We hypothesized that standard measurement of admission BD could improve the existing standards of care and aid clinicians during their initial assessment of patients who might require operative management. Accordingly, we identified 51/84 patients (61%) in the BD ≥ 4 group who required surgical interventions within the first 24 h after injury versus 29/70 patients (41%) in the BD < 4 group (*P* = 0.02; Figure 4(a)). Next, we sought to calculate the relative risk (RR) of requiring surgical intervention in patients presenting to the ED with BD > 4 mEq/L. This analysis revealed that patients in the BD ≥ 4 group have a higher RR of requiring operative management (RR = 1.5, 95% CI [1.056–2.033]; *P* < 0.05) when compared to patients in the BD < 4 group (Figure 4(b)).

Several factors can affect the final disposition of trauma patients, in particular, early prehospital events such as severity of injury sustained and extent of tissue damage/loss after injury [62, 63]. Subsequently, these factors can contribute significantly to the magnitude of the ensuing metabolic stress, which in turn is reflected by high BD values after trauma. Accordingly, we hypothesized that patients admitted to the ED with elevated admission BD could have a differential disposition upon hospital discharge. Our analysis of patients' disposition in both subgroups revealed that the BD ≥ 4 group had greater requirement (*P* = 0.005) for outpatient special care services (72%) when compared to the BD < 4 group (51%) (Table 1).

### 3.5. BD ≥ 4 Group Exhibited a Differential Dynamic Systemic Inflammatory Profile

We next sought to examine the temporal dynamic patterns of circulating inflammation biomarkers between the BD < 4 and BD ≥ 4 subgroups from time of admission and over the 7-day course after injury. Accordingly, three plasma samples were obtained within the first 24 h after injury, including upon arrival as well as daily up to day 7 after injury. To determine difference in levels of inflammatory mediators between the BD < 4 and BD ≥ 4 subgroups, the biomarker data were analyzed using two-way ANOVA (see Section 2). This extensive analysis revealed that the BD ≥ 4 group exhibited statistically significantly higher circulating levels of multiple innate-derived (IL-1RA, IL-6, IL-8/CCL8, MCP-1/CCL2, IFN-*α*, MIP-1*β*/CCL4, and TNF-*α*; see Supplementary Figure  S1 of the Supplementary Material available online at http://dx.doi.org/10.1155/2016/7950374) and lymphoid-derived (IL-7, IL-4, IFN-*γ*, IL-2, IL-13, IL-5, and sIL-2R*α*; Supplementary Figure  S2) inflammatory mediators when compared to the BD < 4 group. However, both subgroups had fairly similar systemic trajectories, with no statistically significant differences, of MIP-1*α*/CCL3, IL-1*β*, IL-15, IL-17A, MIG/CXCL9, IL-10, IP-10/CXCL10, GM-CSF, and Eotaxin/CCL11.

The aforementioned biomarker analysis suggested that the trauma-induced inflammatory response in patients with admission BD ≥ 4 diverges early after injury compared to that of patients with BD < 4. Accordingly, we sought to quantify the total inflammatory mediator production across all time points in the first 24 h by calculating the area under the curve (AUC) in BD < 4 and BD ≥ 4 subgroups. The AUC was calculated for each biomarker and expressed as fold change between both subgroups (see Section 2). Subsequently, the biomarkers were ranked according to their fold values (from highest to lowest fold change). This analysis showed that multiple biomarkers of the innate and lymphoid immune systems were elevated over the first 24 h after injury in the BD ≥ 4 group when compared to the BD < 4 group (Table 2). Interestingly, these biomarkers remained distinct between both groups up to 7 days after injury (Table 3).

### 3.6. Differential Network Connectivity of Systemic Inflammation Inferred in BD ≥ 4 Group

**Figure 5:**
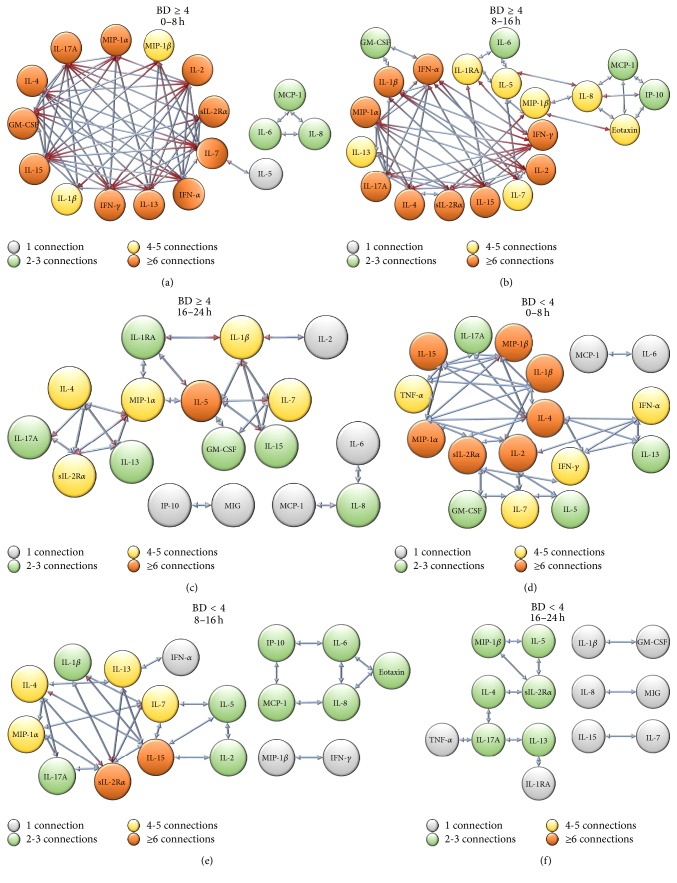
Dynamic network analysis (DyNA) of inflammatory mediators in BD ≥ 4 and BD < 4 subgroups suggests an early differential network connectivity within 16 h after injury. DyNA at 0–8 h suggested that IFN-*α*/sIL-2R*α*/MIP-1*α*/IL-17A/IL-4/GM-CSF/IL-7/IL-2/IFN-*γ*/IL-13 were highly connected in the BD ≥ 4 group (a) while the BD < 4 group (d) exhibited a lesser degree of connected nodes: MIP-1*α*/sIL-2R*α*/MIP-1*β*/IL-4/IL-1*β*/IL-2/IL-15. DyNA at 8–16 h suggested that the BD ≥ 4 group (b) retained the connectivity among inflammatory mediators, whereas the BD < 4 group (e) continued to exhibit a lesser degree of connections. DyNA at 16–24 h suggested that the BD ≥ 4 group (c) exhibited a substantial reduction of network connectivity to a level similar to BD < 4 group (f).

We next hypothesized that these early immune differences between the BD < 4 and BD ≥ 4 subgroups could be explained, at least in part, by differential network interconnectivity among inflammatory mediators. This analysis was achieved by generating networks inferred from correlation changes among circulating inflammatory mediators in a time-dependent manner. To create these dynamic networks, we utilized DyNA (see Section 2), an analysis where we essentially calculate correlations among different inflammatory mediators (nodes) and effectively visualize the temporal interconnectivity of these nodes across multiple time points [17, 56, 57].  Figure 5 shows the detailed DyNA results for BD < 4 and BD ≥ 4 over three different time periods following admission to the ED (0–8 h, 8–16 h, and 16–24 h).

This analysis elucidated that the BD ≥ 4 group exhibited a higher degree of network interconnectivity (≥6 connections) among multiple inflammatory nodes upon admission and up to 16 h after injury (Figures 5(a) and 5(b)). The trauma-induced inflammatory response in the BD ≥ 4 group was characterized by both innate immune (IL-1*β*, GM-CSF, MIP-1*α*/CCL3, MIP-1*β*/CCL4, sIL-1R*α*, IFN-*α*, and IFN-*γ*) and lymphoid (IL-17A, IL-7, IL-4, IL-2, IL-13, and IL-15) pathways. However, the BD < 4 group exhibited a lower degree of network interconnectivity over the initial 0–16 h after injury (Figures 5(d) and 5(e)), with a central network that consisted of IL-4, IL-2, IL-15, MIP-1*α*/CCL3, MIP-1*β*/CCL4, sIL-1R*α*, and IL-1*β*. Interestingly, and by 24 h after injury, both subgroups' network interconnectivity become more comparable and exhibited multiple sparse networks (Figures 5(c) and 5(f)).

**Figure 6:**
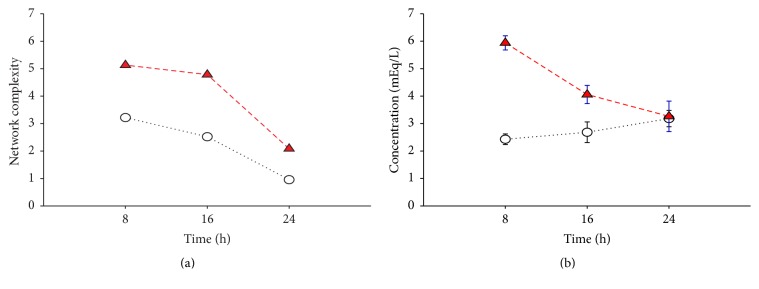
(a) Network complexity differs between the BD ≥ 4 and BD < 4 subgroups over the first 24 h after injury. The BD ≥ 4 group exhibited a higher network density at 8 h and 16 h after injury which progressively decreased to similar levels of BD < 4 group at 24 h after injury. (b) Arterial BD levels within 24 h after injury. BD levels in both BD ≥ 4 and BD < 4 subgroups mirror the density of their corresponding inflammatory networks.

Finally, we sought to assess the global state of the inflammatory networks, by quantifying the total degree of each network present at specific time intervals in both subgroups. The BD ≥ 4 group exhibited a higher network density within 8–16 h after injury when compared to BD < 4 group which then declined progressively over the 16–24 h time period (Figure 6(a)). Interestingly, the inflammation network interconnectivity (*see above*) and density were in parallel with the actual arterial BD values over the first 24 h after injury (Figure 6(b)). Taken together, these analyses suggest a higher, coordinated degree of activation of both innate and lymphoid pathways in patients with admission BD ≥ 4 mEq/L observed as early within the first 8 h after injury.

## 4. Discussion

Severe blunt force trauma can induce substantial physiological changes by alteration of the host metabolic pathways and activation of the immune system [64, 65]. One of the first signs of inadequate tissue perfusion and reduced oxygen delivery is tissue acidosis, which is reflected in serum pH, lactate, arterial BD, and anion gap [66]. Among these parameters, arterial BD has emerged as an important marker in guiding early fluid resuscitation and assessment of shock severity [44, 67, 68]. Despite being sensitive but nonspecific indicator of metabolic acidosis, elevated BD values correlate significantly with in-hospital adverse outcomes [25, 69]. Another key factor that drives outcomes after injury is the magnitude of the accompanying systemic inflammatory response which is largely coordinated by cytokines, chemokines, damage-associated molecular patterns molecules, and free radicals [13, 17].

While a properly robust inflammatory response is essential for effective and timely resolution of the injury, immune dysregulation, on the contrary, can impair both the biochemical and physiological functions and predispose patients to follow a complicated clinical course [17, 70]. Indeed, most evidence suggests that either inadequate [71] or overly exuberant [11] inflammation can drive the pathobiology of trauma and subsequent processes such as persistent critical illness. In view of this, we hypothesized that early, differential immune/inflammatory mechanisms are set in motion by the posttraumatic metabolic acidosis, in a manner associated with distant differential clinical outcomes.

By using a BD cutoff of below versus above 4 mEq/L, we were able to identify and compare two blunt trauma groups with regard to their early dynamic networks of systemic inflammation as well as in-hospital complications as an endpoint of posttraumatic outcome. We initially confirmed key prior observations regarding the association of elevated admission BD with adverse outcomes, namely, significantly greater requirement for blood transfusion, longer ICU and hospital stay, and higher degree of MOD. A key observation from this analysis was that the BD ≥ 4 group had a greater likelihood of requiring operative intervention within the first 24 h after injury.

The BD ≥ 4 and BD < 4 groups did not differ with regard to their overall injury severity, suggesting that the injury severity alone does not appear to be the only driver of metabolic derangement after trauma. Rather, we suggest that the intensity of activation of multiple immune/inflammatory pathways, set in motion early following injury, could drive divergent clinical trajectory in otherwise similarly injured patients. In support of this notion, we have previously shown that distinct, dynamic circulating inflammatory patterns and networks emerge early after trauma, which were associated with in-hospital complications such as nosocomial infection and MOD [17]. In the current study, both groups exhibited a similar elevation in MODScore initially; the degree of MOD persisted in the BD ≥ 4 group but resolved gradually over the 7-day time course in the BD < 4 group, paralleling the divergent systemic inflammatory responses observed in these patient groups. We speculate that these MOD-based immune trajectories are not solely due to metabolic derangement and that other factors such as the injury patterns, specifically extremity injury and subsequent requirement for operative intervention or transfusions, may contribute as well to the early immune divergence after injury.

Analysis of the biomarker patterns showed that the two groups diverged based on IL-6, MCP-1/CCL2, IL-8/CCL8, and IL-1RA as early as we could measure upon admission, correlating with elevated MODScores observed after day 1 and up to day 4 after injury. Prior studies have suggested the utility of elevated circulating inflammation biomarkers, in particular IL-6, as early predictors of MOD in trauma patients [16, 72]. Furthermore, we have previously showed that the chemokine MCP-1/CCL2 plays a central role in driving hepatocyte IL-6 production, as well as systemic IL-6 and outcomes in blunt trauma patients [17, 57, 73].

Though these mediators have been appreciated as being central to the acute inflammatory response, computational modeling has allowed for both their integration into a systems-based process and raising the potential for novel translational applications targeting nonintuitive central mediators as defined by modeling.* In silico* inference of dynamic network interconnectivity and complexity suggests a bifurcation of well-regulated versus dysregulated inflammation in BD < 4 and BD ≥ 4 groups, respectively, early after injury. These findings suggest that the overall magnitude of the inflammatory response is greater in the BD ≥ 4 group and that additional immune components are engaged early in patients subsequent to injury that drives metabolic derangement. Importantly, these* in silico*-defined trajectories of inflammation mimic the clinical trajectories of MOD over the 7 days in both groups, including normalization by ~24 h that is associated with a convergence of BD values in both groups. This normalization may be driven by resuscitation and other supportive measures.

We recognize that there are several limitations in our study. First, this study was performed at a single, Level I trauma center and thus may not be generalizable or pertinent to other centers with differing admission demographics, injury characteristics, or management practices. This issue warrants additional, similar studies in other trauma centers to validate the results suggested from the current study. Another important limitation of this study is the potential impact of blood transfusion and surgical interventions on the temporal dynamics of the inflammatory response. We note that these interventions are by necessity an intrinsic element of clinical care for management of trauma patients with evidence of hypovolemic shock. We suggest that these patients who received this type of interventions reflect patients with hemodynamic and metabolic instability and that by excluding these patients we will be missing the opportunity to capture the early dynamic changes of the inflammatory response. Moreover, the number of inflammatory biomarkers analyzed was limited to the number of analyte we could measure using commercially available Luminex bead sets. Further future studies examining a larger panel of inflammatory biomarkers are suggested. Finally, we note that DyNA lacks mechanistic insight; however, it can be used to understand abstract key features and interactions of the trauma-induced inflammatory response.

## 5. Conclusions

The current study demonstrates the presence of a differential, distinct immune network in the early postinjury phase in BD < 4 and BD ≥ 4 trauma patients which significantly correlate with later divergent clinical outcomes. We suggest that an approach combining extensive early biomarker sampling and computational data-driven modeling can be used as a framework for patient stratification and outcome prediction in the setting of critical illness.

## Supplementary Material

Time course analysis of innate-derived and lymphoid-derived biomarkers between the BD ≥ 4 and BD < 4 subgroups from time of injury up to day 7.

## Figures and Tables

**Table 1:** Overall demographics, clinical outcomes, mechanism of injury, blood and blood components, comorbidities, and disposition of the BD < 4 (*n* = 70) and BD ≥ 4 (*n* = 84) subgroups. Values are expressed as mean ± SEM. Mann–Whitney *U* test, Fisher exact test, or Chi-square were used as appropriate with statistical significance set at *P* < 0.05.

	BD < 4	BD ≥ 4	*P* value
	*n* = 70	*n* = 84	
*Demographics*			
Age, yr	49.3 ± 2.4	45.76 ± 2	0.34
Sex, male/female	M = 55 F = 15	M = 55 F = 29	0.07
Injury severity score (ISS)	21 ± 1.3	23 ± 1.2	0.15
Glasgow coma scale (GCS)	12.7 ± 0.52	12.6 ± 0.56	0.46
*Outcome*			
Intensive Care Unit length of stay, days	6.6 ± 0.79	9.9 ± 0.96	0.005
Hospital length of stay, days	11.9 ± 0.97	17.4 ± 1.2	<0.001
Mechanical ventilation, days	3 ± 0.56	5.5 ± 0.87	0.02
*Mechanism of Injury*			
Motor vehicle crashes (MVC), *n* (%)	47 (67%)	55 (65%)	0.98
Fall, *n* (%)	8 (11.5%)	10 (12%)	
Motorcycle, *n* (%)	8 (11.5%)	9 (11%)	
Others, *n* (%)	7 (10%)	10 (12%)	
*Blood and Blood Components*			
24 h packed RBC, *n* (%)	10 (14.3%)	27 (32.2%)	0.001
24 h blood components (platelets, FFP, and cryoprecipitate), *n* (%)	4 (5.7%)	0 (0%)	
24 h PRBC + other components (platelets, FFP, and cryoprecipitate), *n* (%)	6 (8.6%)	16 (19%)	
None, *n* (%)	50 (71.4%)	41 (48.8%)	
*Comorbid Conditions*			
Hypertension, *n* (%)	22 (31.4%)	20 (23.8%)	0.3
Diabetes mellitus, *n* (%)	10 (14.3%)	9 (10.7%)	0.5
Smoking, *n* (%)	4 (5.7%)	6 (7.2%)	0.7
Cardiovascular, *n* (%)	10 (14.3%)	12 (14.3%)	1
Pulmonary, *n* (%)	3 (4.3%)	8 (9.5%)	0.2
Endocrine, *n* (%)	15 (21.4%)	8 (9.5%)	0.04
Gastrointestinal, *n* (%)	6 (8.6%)	5 (6%)	0.5
Peripheral vascular disease, *n* (%)	2 (2.9%)	0 (0%)	0.12
Neurological, *n* (%)	8 (11.4%)	4 (4.8%)	0.12
Psychiatric, *n* (%)	6 (8.6%)	9 (10.7%)	0.65
Drug and Alcohol Abuse, *n* (%)	4 (5.7%)	4 (4.8%)	0.79
Renal, *n* (%)	0 (0%)	4 (4.8%)	0.06
Rheumatologic, *n* (%)	0 (0%)	4 (4.8%)	0.06
Hematologic, *n* (%)	3 (4.3%)	1 (1.2%)	0.23
Previous admission for trauma, *n* (%)	0	1 (1.2%)	0.36
None, *n* (%)	22 (31.4%)	32 (38.1%)	0.39
*Disposition*			
Home, *n* (%)	34 (48.6%)	23 (27.4%)	0.005
Home with service, *n* (%)	2 (2.9%)	1 (1.2%)	
Inpatient rehabilitation facility, *n* (%)	6 (8.6%)	1 (1.2%)	
Outpatient rehabilitation facility, *n* (%)	11 (15.7%)	17 (20.2%)	
Skilled nursing facility, *n* (%)	16 (22.9%)	39 (46.4%)	
Others, *n* (%)	1 (1.4%)	3 (3.6%)	

**Table 2:** Summary of the area under the curve (AUC) analysis in the first 24 h after injury for statistically significantly different inflammatory mediators (by two-way ANOVA) between the BD < 4 and BD ≥ 4 subgroups.

Inflammation biomarkers (0–24 h after injury)	BD ≥ 4 (pg.h/L)	BD < 4 (pg.h/L)	Fold change	*P* value
IL-1RA	2268.0	728.3	3.1	<0.001
IL-7	208.0	94.6	2.2	0.036
IL-8/CCL8	222.3	103.7	2.1	0.004
sIL-2R*α*	711.2	373.8	1.9	0.005
MCP-1/CCL2	2336.7	1423.3	1.6	0.048

**Table 3:** Summary of the area under the curve (AUC) analysis from the time of injury up to day 7 for statistically significantly different inflammatory mediators (by two-way ANOVA) between the BD < 4 and the BD ≥ 4 subgroups.

Inflammation biomarkers (0 h–day 7 after injury)	BD ≥ 4 (pg.h/L)	BD < 4 (pg.h/L)	Fold change	*P* value
IL-1RA	7409.3	3731.0	2.0	<0.001
IL-5	276.1	150.4	1.8	0.005
IL-7	880.2	479.5	1.8	0.001
sIL-2R*α*	4026.7	2356.0	1.7	<0.001
IL-6	1648.9	1022.5	1.6	0.013
MCP-1/CCL2	7254.3	4747.9	1.5	<0.001
IFN-*γ*	485.0	332.5	1.5	0.002
IL-13	223.5	159.9	1.4	0.002
IL-4	466.3	342.1	1.4	0.002
IL-8/CCL8	546.7	401.6	1.4	0.045
IFN-*α*	575.3	430.6	1.3	<0.001
IL-2	100.8	79.6	1.3	0.037
MIP-1*β*/CCL4	993.0	789.2	1.3	0.029
TNF-*α*	115.9	94.2	1.2	0.003

## Abbreviations

MOD: Multiple organ dysfunction
ICU: Intensive Care Unit
ED: Emergency Department
DyNA: Dynamic network analysis
IL: Interleukin
IFN: Interferon
MCP-1/CCL2: Monocyte chemotactic protein-1
MIG/CXCL-9: Monokine inducible by interferon-*γ*
MIP-1*α*/CCL-3: Macrophage inflammatory protein-1*α*.
