# Supplementary material for: Elevated Admission Base Deficit Is Associated with a Complex Dynamic Network of Systemic Inflammation Which Drives Clinical Trajectories in Blunt Trauma Patients
Source: Mediators Inflamm. 2016 Nov 15;2016:7950374. doi: 10.1155/2016/7950374 (PMC5126463; doi:10.1155/2016/7950374)
Supplement: Supplementary file 1 — Time course analysis of innate-derived and lymphoid-derived biomarkers between the BD ≥ 4 and BD < 4 subgroups from time of injury up to day 7. [file 7950374.f1.pdf]

Supplementary Material

Fig. S1 Innate Inflammatory Mediators

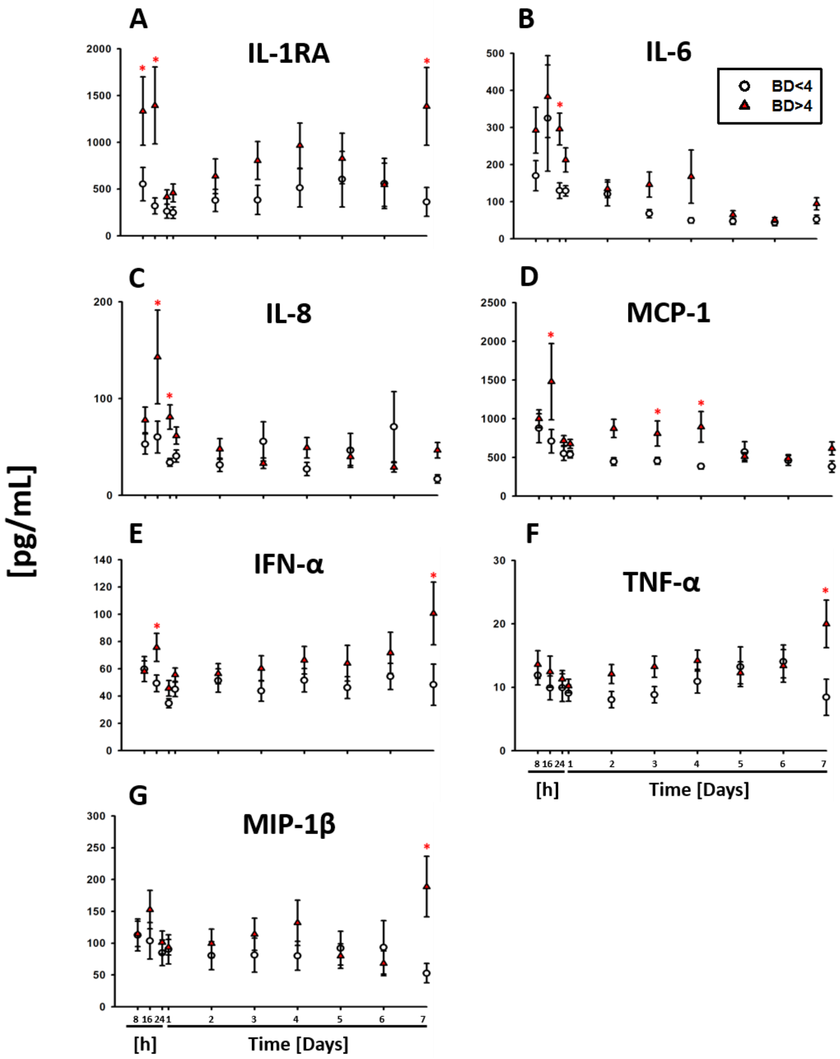

Figure S1. Time course analysis of innate-derived biomarkers between the BD ≥ 4 and BD < 4 sub-groups from time of injury up to day 7. Mean circulating levels of inflammatory mediators in BD ≥ 4 (n = 84) vs. BD < 4 (n = 70). (**A**) Time course of IL-1RA. (**B**) Time course of IL-6. (**C**) Time course of IL-8. (**D**) Time course of MCP-1. (**E**) Time course IFN-α. (**F**) Time course of TNF-α. (**G**) Time course of MIP-1β. The indicated inflammatory mediators were assessed in serial plasma samples obtained at the indicated time points. Values are mean ± SEM. **P* < 0.05 by Two-Way ANOVA.

Fig. S2 Lymphoid Inflammatory Mediators

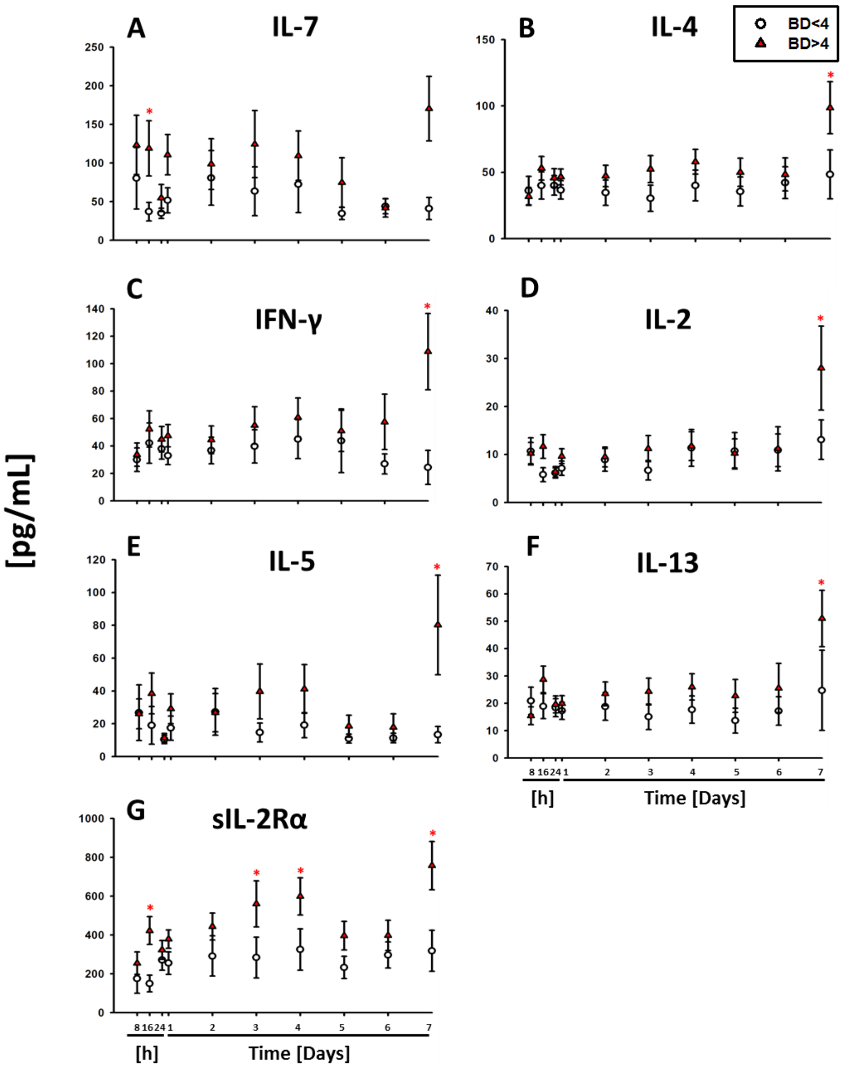

Figure S2. Time course analysis of lymphoid-derived biomarkers between the BD ≥ 4 and BD < 4 sub-groups from time of injury up to day 7. Mean circulating levels of inflammatory mediators in BD ≥ 4 (n = 84) vs. BD < 4 (n = 70). (**A**) Time course of IL-7. (**B**) Time course of IL-4. (**C**) Time course of INF-γ. (**D**) Time course of IL-2. (**E**) Time course IL-5. (**F**) Time course of IL-13. (**G**) Time course of sIL-2Rα. The indicated inflammatory mediators were assessed in serial plasma samples obtained at the indicated time points. Values are mean ± SEM. *$P < 0.05$ by Two-Way ANOVA.
